# Supplementary material for: Molecular Interactions of the Copper Chaperone Atx1 of Paracoccidioides brasiliensis with Fungal Proteins Suggest a Crosstalk between Iron and Copper Homeostasis
Source: Microorganisms. 2023 Jan 18;11(2):248. doi: 10.3390/microorganisms11020248 (PMC9963772; doi:10.3390/microorganisms11020248)
Supplement: Supplementary file 1 [file microorganisms-11-00248-s001.zip › microorganisms-2087477-supplementary.pdf]

```
PbAtx1      -------------MASVEHQYKFNISMSCGGCSGAVERVLKKLD-GVKSYTVNLESQTAT    46
ScAtx1      --------------MAEIKHYQFNVVMTCSGCSGAVNKVLTKLEPDVSKIDISLEKQLVD    46
CnAtx1      MAAPGTIITSTPGAVPAGPAYQYSVKMTCTGCSGAINRVLGKNITAPNAYHISLPKQLVL    60
AfAtxA      ---------------MSEHQYKFNVSMSCGGCSGAVERVLKRLD-GVKSFDVNLDSQTAL    44
                                *::.: *:* *****:::** :     .   :.* .* .

PbAtx1      VVADPSLEYDTVLSTIKKTGKTVNSGEADGEPKDV-------    81
ScAtx1      VYTT--LPYDFILEKIKKTGKEVRSGKQ--------------    72
CnAtx1      VWGPSLPPFETVTEKIAKTGKTINAKEVIEDASTLPPLEAAA    102
AfAtxA      VTTEPTVSYETVLATIKKTGKTVNSGEADGKPMDV-------    79
            *       :: :  .* **** :.: :
```

Supplementary Figure S1: Conserved copper-binding motif in fungal Atx. Amino acid alignment of Atx1 demonstrating the conservation of copper-binding motif (*MXCXXC*) among fungal species. Purple: methionine residues. Green: cysteine residues. Asterisks represents amino acid identity and dots indicates conserved substitutions. Accession numbers: *Pb* (PADG_02352), *Sc* (YNL259C), *Cn* (CNE01230), *Af* (Afu1g08880).

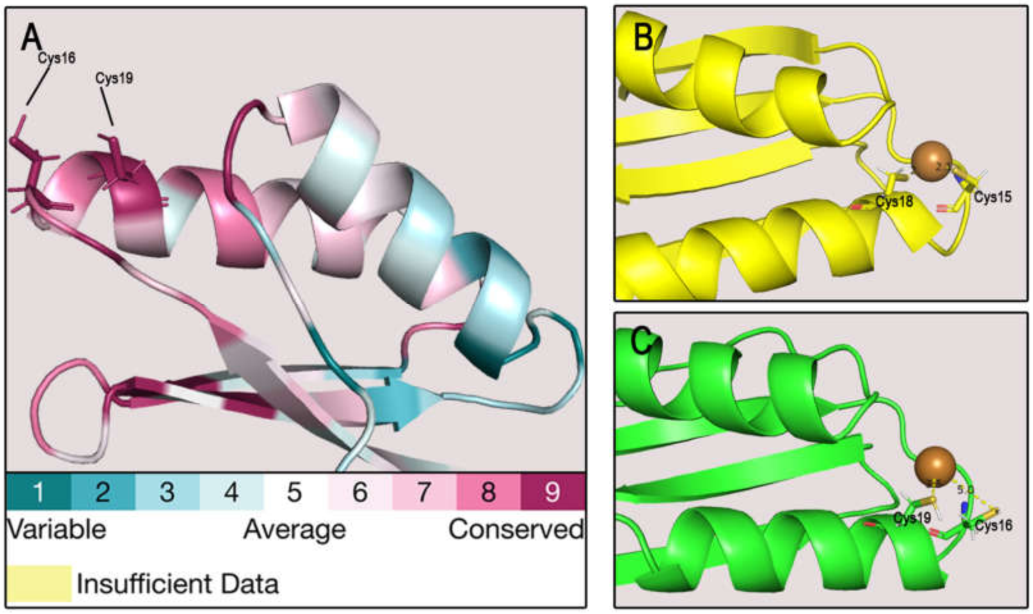

Supplementary Figure S2: Evolutionary conservation of PbAtx1 and copper binding motif comparisons. (**A**)ConSurf analysis showing the conservation level of PbAtx1 amino acid residues. It is possible to perceive the high degree of conservation of the cysteine residues (Cys16 and Cys19) that compose the predicted copper-binding motif. (**B**)Copper binding mode to Cys15 and Cys18 residues in the crystallographic structure of ScAtx1p (PDB Entry ID: 1FD8) showing distances of 2.1 Å for the hydrogen bonds in this interaction. (**C**)Prediction of copper binding to Cys16 and Cys19 residues in PbAtx1 showing distances of 5.0 Å and 3.5 Å for hydrogen bonds respectively.
